# SOXs in human prostate cancer: implication as progression and prognosis factors

**DOI:** 10.1186/1471-2407-12-248

**Published:** 2012-06-15

**Authors:** Wei-de Zhong, Guo-qiang Qin, Qi-shan Dai, Zhao-dong Han, Shan-ming Chen, Xiao-hui Ling, Xin Fu, Chao Cai, Jia-hong Chen, Xi-bin Chen, Zhuo-yuan Lin, Ye-han Deng, Shu-lin Wu, Hui-chan He, Chin-lee Wu

**Affiliations:** 1 Department of Urology, Guangdong Key Laboratory of Clinical Molecular Medicine and Diagnostics, Guangzhou First Municipal People’s Hospital, Affiliated Guangzhou Medical College, Guangzhou, 510180, China; 2 Urology Key Laboratory of Guangdong Province, Guangzhou Medical College, Guangzhou, 510230, China; 3 Guangdong Provincial Institute of Nephrology, Southern Medical University, Guangzhou, 510515, China; 4 Departments of Pathology and Urology, Massachusetts General Hospital, Harvard Medical School, Boston, MA, USA

**Keywords:** Prostate cancer, SOX, Clinicopathological feature, Biochemical recurrence-free survival

## Abstract

**Background:**

SOX genes play an important role in a number of developmental processes. Potential roles of SOXs have been demonstrated in various neoplastic tissues as tumor suppressors or promoters depending on tumor status and types. The aim of this study was to investigate the involvement of SOXs in the progression and prognosis of human prostate cancer (PCa).

**Methods:**

The gene expression changes of SOXs in human PCa tissues compared with non-cancerous prostate tissues was detected using gene expression microarray, and confirmed by real-time quantitative reverse transcriptase-polymerase chain reaction (QRT-PCR) analysis and immunohositochemistry. The roles of these genes in castration resistance were investigated in LNCaP xenograft model of PCa.

**Results:**

The microarray analysis identified three genes (SOX7, SOX9 and SOX10) of SOX family that were significantly dis-regulated in common among four PCa specimens. Consistent with the results of the microarray, differential mRNA and protein levels of three selected genes were found in PCa tissues by QRT-PCR analysis and immunohistochemistry. Additionally, we found that the immunohistochemical staining scores of SOX7 in PCa tissues with higher serum PSA level (*P* = 0.02) and metastasis (*P* = 0.03) were significantly lower than those with lower serum PSA level and without metastasis; the increased SOX9 protein expression was frequently found in PCa tissues with higher Gleason score (*P* = 0.02) and higher clinical stage (*P* < 0.0001); the down-regulation of SOX10 tend to be found in PCa tissues with higher serum PSA levels (*P* = 0.03) and advanced pathological stage (*P* = 0.01). Moreover, both univariate and multivariate analyses showed that the down-regulation of SOX7 and the up-regulation of SOX9 were independent predictors of shorter biochemical recurrence-free survival. Furthermore, we discovered that SOX7 was significantly down-regulated and SOX9 was significantly up-regulated during the progression to castration resistance.

**Conclusions:**

Our data offer the convince evidence that the dis-regulation of SOX7, SOX9 and SOX10 may be associated with the aggressive progression of PCa. SOX7 and SOX9 may be potential markers for prognosis in PCa patients. Interestingly, the down-regulation of SOX7 and the up-regulation of SOX9 may be important mechanisms for castration-resistant progression of PCa.

## Background

Prostate cancer (PCa) is one of the most prevalent malignancies in men and the second most frequent cause of male cancer-related death [1]. It is a clinically heterogeneous-multifocal disease and the incidence is continuously rising. Carcinogenesis and the mechanisms influencing the progression and prognosis of PCa is a multistep process, involving both genetic insults to epithelial cells and changes in epithelial-stromal interactions. In spite of current therapeutic methods, many patients develop metastases. Androgen suppression therapies (ASTs) have already been considered as the mainstay of systemic treatment for advanced PCa, especially for metastatic disease [2]. Most patients respond to AST and their clinical symptoms could be relieved, although they eventually experience disease relapse and develop more aggressive tumors, commonly termed 'hormone-refractory prostate cancer' or 'castration-resistant prostate cancer' [3]. The clinical benefits of AST for various PCa patients are different. Even among patients with metastatic PCa, for which AST is the established standard of care, the length of clinical remission induced by AST can range from months to more than 10 years [4]. Although some clinical parameters, such as serum prostate specific antigen (PSA) levels, may provide some prognostic utility in the treatment settings, there are currently no definitive clinical methods that can reliably predict the responses to clinical therapies for PCa. Thus, it is of great significance to search for a more sensitive and more specific PCa marker that can provide valuable information for the diagnosis and treatment of the disease.

SOX genes belong to the High Mobility Group (HMG) superfamily and encode transcription factors which can be identified based on a conserved motif within the HMG domain, RPMNAFMVW [5]. It has been demonstrated that the SOX genes can regulate a number of developmental processes, including lens, hair follicle, gut, B-cell, muscle, and blood vessel development [6]. Their potential roles have also been found in various neoplastic tissues as tumor suppressors or promoters depending on tumor status and types. Of our interests, Wang et al. [7,8] found that SOX9 is expressed in primary PCa in vivo, at a higher frequency in recurrent PCa and in PCa cell lines. They also demonstrated that the suppression of SOX9 by siRNA in PCa cells may reduce endogenous androgen receptor (AR) protein levels and cell growth, which suggested that SOX9 may contribute to androgen receptor regulation and decreased cellular proliferation of PCa. In addition, Katoh et al. [9,10] detected the SOX7 expression in various normal and tumor tissues. They found that SOX7 mRNA was relatively highly expressed in a gastric cancer and esophageal cancer cell lines, but was significantly down-regulated in primary colorectal tumor, breast cancer, kidney tumor, lung cancer and PCa tissues.

Perplexing expression patterns of SOXs in PCa have not been clear. To address this problem, in our studies, SOX7, SOX9 and SOX10 were identified as target genes because of their statistically differential expression in PCa tissues detected by the gene microarray. In addition, expression states of the mRNA and protein of these candidate genes were evaluated further by real-time quantitative reverse transcriptase-polymerase chain reaction (QRT-PCR) analysis, and semi-quantitative immunohistochemistry in a large series of PCa and adjacent benign prostate tissues. Then, the clinicopathological relevance and prognostic value of SOX7, SOX9 and SOX10 proteins have also been determined. Furthermore, the roles of SOX7, SOX9 and SOX10 genes in castration resistance were investigated in a LNCaP xenograft model of PCa.

## Methods

### Patients and tissue samples

The study was approved by the Research Ethics Committee of Guangzhou First Municipal People’s Hospital, Affiliated Guangzhou Medical College, China. Informed consent was obtained from all of the patients. All specimens were handled and made anonymous according to the ethical and legal standards.

For gene microarray analysis, 4 pairs of prostate primary tumor and benign tissues adjacent to tumor were collected from the tissue bank at Guangzhou Medical College. The detail information on these tissues has been described in our previous study [11].

For QRT-PCR analysis, 10 fresh PCa tissues (aging 54 ~ 87 years, mean ± SD = 73.9 ± 10.9 years, TNM staging from I to III) and paired 10 adjacent benign tissues of prostate were provided by the Guangzhou First Municipal People’s Hospital. Among them, 4 pairs of samples are the same with gene microarray analysis.

For semiquantitative immunohistochemistry, tissue microarray was provided by the Massachusetts General Hospital. There include 147 PCa tissues (aging 37 ~ 83 years, mean ± SD = 58.2 ± 7.0 years, TNM staging from I to III) and 28 paired adjacent benign tissues.

All of the tissues were obtained immediately during the operation of transurethral resection prostate and suprapubic prostatectomy. None of the patients recruited in this study had chemotherapy or radiotherapy before the surgery. The pathological diagnosis was performed preoperatively and confirmed postoperatively. All patients were reviewed and all specimens were re-examined in March, 2010. TMN grade and Gleason Score were examined by the same group of two senior pathologists experienced in PCa diagnosis. The detail information on the clinical features of all patients in this study was shown in Table 1.

**Table 1:** Clinical features of all patients

**Sample type & Clinical features**	**Experiment type (cases)**		
	**Microarray**	**RT-QPCR**	**IHC**
Prostate cancer	4	10	147
Mean age (range, years)	70.25 ± 11.6154	73.90 ± 10.8572	58.17 ± 7.038
	(54-80)	(54-87)	(37-83)
≤60	1	2	99
>60	3	8	48
Serum PSA levels (ng/ml)			
<10	1	1	10
≥10	3	9	74
Gleason score			
<8	2	4	29
≥8	2	6	118
Metstasis	0	0	19
Adjacent benign prostate tissue	4	10	28

The patients involved in the semiquantitative immunohistochemistry were given a follow-up exam ranging from 3 to 12 years. For the analysis of survival and follow-up, the date of prostatectomy was used to represent the beginning of the follow-up period. The primary analysis endpoint was biochemical recurrence-free survival. Others analysis endpoints were overall survival and metastasis-free survival. All the patients who died from diseases other than PCa or from unexpected events were excluded from the case collection.

### Animals and xenograft tumor establishment

All experiments involving laboratory animals were done in accordance with the Guideline for Animal Experiments of Guangzhou Medical College and approved by Animal Research Committee at Guangzhou Medical College. 6–8 weeks old male athymic mice (BALB/c strain, Charles River Laboratory, Montreal, PQ, Canada) were used.

Early passage LNCaP cells were grown in RPMI 1640 (Life Technologies) with 10% fetal bovine serum (FBS), 100 units/mL penicillin, and 100 μg/mL streptomycin under 5% CO_2_. Cells underwent 4-8 passages prior to mouse inoculation. Approximately 10^6^ LNCaP cells suspended in 250 μl media were mixed with 250 μl Matrigel (Becton Dickinson labware, Bedford, MA) and then inoculated subcutaneously in the flank region of athymic mice using a 25-gauge needle.

### Treatment protocol and tissue sampling

Once established at 8-12 weeks after injection, tumors were measured biweekly. Tumor volumes were measured with a caliper using the formula: length × width^2^ × 0.52. The mice bearing xenograft tumors were castrated after the xenograft volume reached 150 mm^3^. The sequential changes in tumor volume were analyzed. Serum samples were obtained at sacrifice to measure PSA values using a sandwich enzyme immunoassay kit (CAN-tPSA-4300, Diagnostics Biochem Canada Inc., Ontario, Canada) with a lower limit of detection of 0.1 ng/ml. Xenograft tissues of mice were collected during various stages and total RNA was isolated and purified using the RNeasy Mini Kit (Qiagen).

### Gene microarray analysis

The gene microarray analysis has been reported by our previous study [11] and the microarray raw data have been depostied at GEO under the accession number GSE28204.

### Real-time quantitative reverse transcriptase PCR

Quantitative PCR was used to examine the expression status of the three candidate genes which have statistically differential expression in PCa tissues compared with non-cancerous prostate tissues detected by gene microarray analysis.

The cDNA templates for qRT-PCR were synthesized from RNA samples. The primers 5'- TGA GCC AGG TGG AAC TCC T -3' and 5'- CTG GGA GAC CGG AAC ATG C -3' were used to amplify 125-bp transcripts of SOX7, the primers 5'- AGC GAA CGC ACA TCA AGA C -3' and 5'- GCT GTA GTG TGG GAG GTT GAA -3' were used to amplify 110-bp transcripts of SOX9, the primers 5'- CCC ACA CTA CAC CGA CCA G -3' and 5'- GGC CAT AAT AGG GTC CTG AGG -3' were used to amplify 143-bp transcripts of SOX10, and the primers 5'- GGT GGC TTT TAG GAT GGC AAG -3' and 5'- ACT GGA ACG GTG AAG GTG ACA G -3' were used to amplify 161-bp transcripts of β-actin. Gene expression was determined using SYBR Green PCR mix (Toyoko) and 1 μg of template. Real-time PCR was performed on a MyiQ.2 Two-Color Real-Time PCR Detection System (Bio-Rad), using the following amplification conditions: 5 min, 95°C; followed by 40 cycles of 10 seconds 95°C, 20 seconds 60°C and 20 seconds 72°C. All assays were carried out in triplicate. CT-values were determined using the IQ5 software (Bio-Rad). Gene expression in each sample was normalized with the housekeeping gene (β-actin) expression. Relative quantification of target gene expression was evaluated using the comparative cycle threshold (CT) method.

### Immunohistochemistry analysis

The specimens were fixed in 10% neutral buffered formalin and subsequently embedded in paraffin. The paraffin-embedded tissues were cut at 3 μm and then deparaffinized with xylene and rehydrated for further H&E or peroxidase (DAB) immunohistochemistry staining employing DAKO EnVision System (Dako Diagnostics, Zug, Switzerland). Briefly, following a brief proteolytic digestion and a peroxidase blocking of tissue slides, the slides were incubated overnight with the primary antibodies against respective target proteins (SOX7 Ab., Santa Cruz #sc-20093; SOX9 Ab., Santa Cruz #sc-20093; SOX10 Ab., Santa Cruz # sc-17342) at a dilution of 1:50 (SOX7 and SOX10) and 1:100(SOX9) at 4°C. After washing, peroxidase labeled polymer and substrate-chromogen were then employed in order to visualize the staining of the interested proteins. In each immunohistochemistry run, negative controls were carried out by omitting the primary antibody, whereas the overexpressions of respective target proteins confirmed by western blotting was used as positive controls.

Following a hematoxylin counterstaining, immunostaining was scored by two independent experienced pathologists, who were blinded to the clinicopathological data and clinical outcomes of the patients. The scores of the two pathologists were compared and any discrepant scores were trained through re-examining the stainings by both pathologists to achieve a consensus score. The number of positive-staining cells in ten representative microscopic fields was counted and the percentage of positive cells was calculated. Given the homogenicity of the staining of the target proteins, tumor specimens were scored in a semi-quantitative manner. The percentage scoring of immunoreactive tumor cells was as follows: 0 (0%), 1 (1-10%), 2 (11-50%) and 3 (>50%). The staining intensity was visually scored and stratified as follows: 0 (negative), 1 (weak), 2 (moderate) and 3 (strong). A final immunoreactivity scores (IRS) was obtained for each case by multiplying the percentage and the intensity score. Protein expression levels were further analyzed by classifying IRS values as low (based on a IRS value less than 4) and as high (based on a IRS value greater than 4).

### Western blot analysis

For western blot analysis, 40 μg of whole cell extracts were fractioned by SDS-PAGE and transferred onto Hybond nitrocellulose membranes (GE Healthcare). Filters were blocked in PBS-Tween 20/5% skim milk and probed with antibodies against respective target proteins (SOX7 Ab., Santa Cruz #sc-20093; SOX9 Ab., Santa Cruz #sc-20093; SOX10 Ab., Santa Cruz # sc-17342) at a dilution of 1:50 (SOX7 and SOX10) and 1:100 (SOX9), or probed with anti-β-catenin antibodies, which were visualized by SuperSignal West PICO chemiluminescent detection system (Pierce Biotechnology). β-actin was used as equal protein loading control.

### Statistical analysis

The software of SPSS version13.0 for Windows (SPSS Inc, IL, USA) and SAS 9.1 (SAS Institute, Cary, NC) was used for statistical analysis. Continuous variables were expressed as $\overset{―}{X}\pm s$. Statistical analysis were performed with Fisher's exact test for any 2 × 2 tables,Pearson χ^2^ test for non-2 × 2 tables, Kaplan-Meier method for the question of survival, Chiquest trend test for ordinal data, and Cox regression analysis for the multivariate analysis. The Spearman correlation was calculated between the expression levels of SOX7, SOX9 and SOX10 in PCa tissues. Differences were considered statistically significant when *p* was less than 0.05.

## Results

### Identification of candidate target SOX genes for PCa by gene microarray analysis

In the search for SOX genes involved in the development of human PCa, we compared gene expresion between 4 pairs of PCa and adjacent benign prostate tissue samples. As the results, three SOX genes--SOX7 (*p* < 0.001) and SOX10 (*p* = 0.010) were down-regulated, but SOX9 (*p* = 0.029) was up-regulated in all PCa tissue samples (Table 2).

**Table 2:** Microarray data of SOX family genes

**Gene Symbol**	**Gene_ID**	**regulation**	**Fold_change**	***p*-value**
SOX1	6656	up	1.19	0.358
SOX2	6657	down	4.37	0.146
SOX3	6658	down	1.29	0.353
SOX4	6659	up	1.77	0.435
SOX5	6660	down	2.11	0.137
SOX6	55553	down	1.23	0.358
**SOX7**	**83595**	**down**	**2.11**	**<0.001**
SOX8	30812	up	1.44	0.404
**SOX9**	**6662**	**up**	**1.57**	**0.029**
**SOX10**	**6663**	**down**	**1.69**	**0.010**
SOX11	6664	up	1.82	0.226
SOX12	6666	up	1.99	0.399
SOX13	9580	down	1.25	0.461
SOX14	8403	up	1.03	0.055
SOX15	6665	up	1.42	0.271
SOX17	64321	up	1.35	0.161
SOX18	54345	up	1.22	0.407
SOX21	11166	up	1.62	0.053
SOX2OT	347689	down	1.59	0.248
SOX30	11063	up	1.21	0.504

Note: Genes marked with grey color were significantly differentially expressed in PCa tissues compared with non-cancerous prostate tissues.

### Validation of the gene microarray results by real-time quantitative RT-PCR

**Figure 1:**
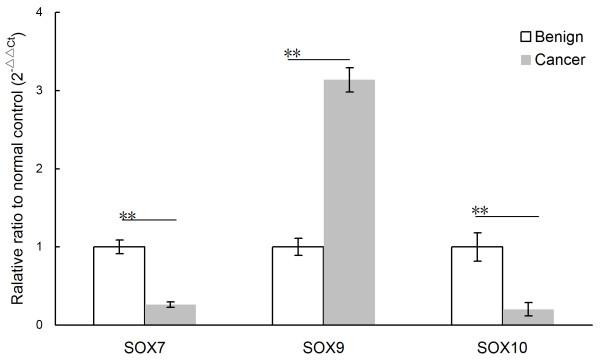
**Validation of gene microarray data by real-time quantitative RT-PCR (qRT-PCR).** Three genes were subjected to QRT-PCR in the mRNA from ten pairs of PCa and adjacent benign prostate tissues. The significant down-regulation of SOX7 and SOX10 and up-regulation of SOX9 were detected in PCa tissues (n = 10) than that in the corresponding adjacent benign prostate tissues (all *P* < 0.01). Relative expression ratio is defined as the expression levels of the gene to those of the internal reference gene, β-actin. The assays were carried out in triplicate and means ± standard deviations were plotted.

To further validate the results of gene microarray analysis, we performed QRT-PCR to detect the mRNA expression levels of SOX7, SOX9 and SOX10 in 10 PCa and 10 adjacent benign prostate tissues. We found that SOX7 (*p* < 0.01) and SOX10 (*p* < 0.01) genes showed a significant down-regulation in tumor tissues and SOX9 (*p* < 0.01) gene showed a significant up-regulation in tumor tissues when compared to corresponding adjacent benign prostate tissues, although the fold change in the expression level was not exactly same between the microarray analysis and QRT-PCR (Figure 1).

### Expression of SOX7, SOX9 and SOX10 proteins in PCa and adjacent benign prostate tissues

**Figure 2:**
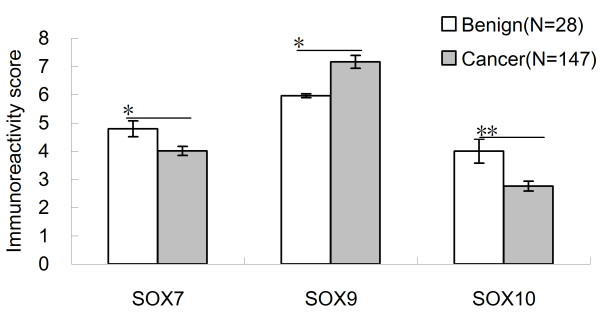
Immunoreactivity scores (IRS) of SOX7, SOX9 and SOX10 in PCa and Benign tissues.^*^p < 0.05 and ^**^p < 0.001 in comparison between the IRS of three proteins in PCa and Benign tissues.

**Figure 3:**
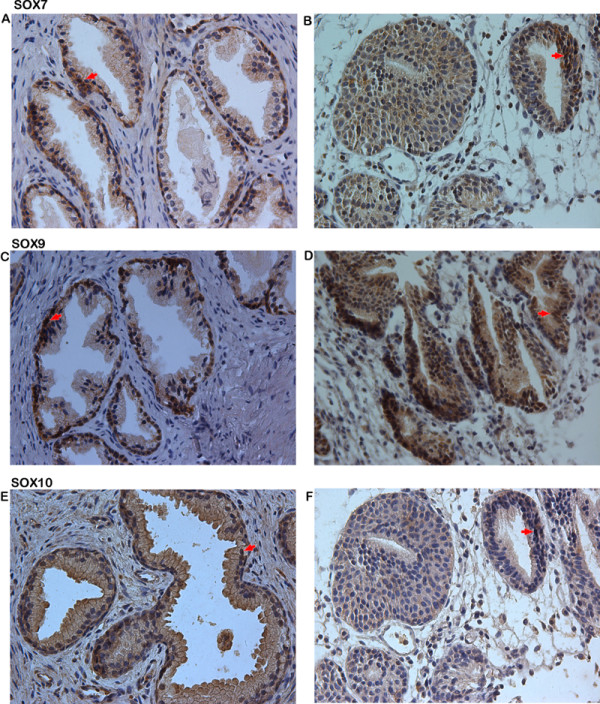
**Immunohistochemical staining for SOX7, SOX9 and SOX10 in PCa (Original magnification × 200). a,** SOX7 strongly positive staining was found in cytoplasm of Benign tissue; **b,** SOX7 weakly positive staining in PCa was found in cytoplasm of PCa tissues; **c,** SOX9 weakly positive staining was found in nucleus of Benign tissue; **d,** SOX9 strongly positive staining in PCa was found in nucleus of PCa tissues; **e,** SOX10 strongly positive staining was found in cytoplasm of Benign tissue; **f,** SOX10 weakly positive staining in PCa was found in cytoplasm of PCa tissues.

The expression and localization of SOX7, SOX9 and SOX10 in the 147 PCa and 28 adjacent benign prostate tissues were examined using immunohistochemical analysis. The expression levels of SOX7 (IRS: PCa = 4.01 ± 0.160 vs. Benign = 4.79 ± 0.279, *p* = 0.024) and SOX10 (IRS: PCa = 2.76 ± 0.174 vs. Benign = 4.00 ± 0.424, *p* = 0.010) in PCa tissues were lower than those in adjacent benign prostate tissues significantly (Figure 2). But the expression level of SOX9 in PCa tissues were higher than that in adjacent benign prostate tissues (IRS: PCa = 7.16 ± 0.228 vs. Benign = 5.96 ± 0.461, *p* = 0.041) significantly (Figure 2). The SOX7 and SOX9 expression occurred mainly in the nucleus, while SOX10 in the cytoplasm, which is similar to the results from the previous studies [10,12]. Representative pictures of immunohistochemistry staining of SOX7, SOX9 and SOX10 are shown in Figure 3. In addition, the Spearman Correlation analysis showed that the expression level of SOX7 protein in PCa tissues was significantly negative correlated with that of SOX9 protein (rs = -0.408, *p* < 0.001) and positive correlated with that of SOX10 protein (rs = 0.513, *p* < 0.001), respectively; the expression level of SOX9 protein was also significantly negative correlated with that of SOX10 protein (rs = -0.219, *p =* 0.008).

### Association of SOX7, SOX9 and SOX10 expression with the clinicopathological characteristics of PCa

The association of SOX7, SOX9 and SOX10 expression with the clinicopathological features of PCa patients is shown in Table 3. The immunohistochemical staining scores of SOX7 in PCa tissues with higher serum PSA level (*P* = 0.021) and metastasis (*P* = 0.025) were significantly lower than those with lower serum PSA level and without metastasis; the increased SOX9 protein expression was frequently found in PCa tissues with higher Gleason score (*P* = 0.024) and higher clinical stage (*P* < 0.0001); the down-regulation of SOX10 tend to be found in PCa tissues with higher serum PSA levels (*P* = 0.025) and advanced pathological stage (*P* = 0.013).

**Table 3:** Relationship between immunoreactivity scores of SOX7, SOX9, SOX10 and clinicopathological features of PCa

**Clinical features (Numbers)**	**SOX7**		**SOX9**		**SOX10**	
	**IRS**	***P***	**IRS**	***P***	**IRS**	***P***
	***X̅* ± *s***		***X̅* ± *s***		***X̅* ± *s***	
Age (years)						
< 70 (75)	3.93 ± 0.219	0.530	7.46 ± 0.268	0.114	2.73 ± 0.215	0.875
≥70 (72)	4.14 ± 0.224		6.68 ± 0.403		2.79 ± 0.295	
Serum PSA Levels (ng/ml)						
<4 (27)	4.50 ± 0.330	0.021	7.17 ± 0.560	0.968	3.29 ± 0.456	0.025
≥4 (120)	3.34 ± 0.183		7.14 ± 0.251		2.33 ± 0.185	
Gleason Score						
< 8 (118)	4.09 ± 0.183	0.317	5.93 ± 0.606	0.024	2.66 ± 0.188	0.276
≥8 (29)	3.69 ± 0.322		7.46 ± 0.235		3.14 ± 0.438	
Clinical Stage						
<T2A(79)	3.95 ± 0.205	0.431	6.39 ± 0.326	<0.001	2.62 ± 0.226	0.498
≥T2A(68)	4.21 ± 0.259		8.25 ± 0.270		2.86 ± 0.269	
Pathological Stage						
T2A-T2C(86)	4.06 ± 0.209	0.742	7.23 ± 0.282	0.693	3.53 ± 0.214	0.013
T3A-T4(61)	3.95 ± 0.249		7.05 ± 0.381		2.27 ± 0.287	
Metstasis						
No (119)	4.88 ± 0.179	0.025	7.34 ± 0.240	0.104	2.67 ± 0.186	0.327
Yes (28)	3.75 ± 0.359		6.39 ± 0.611		3.11 ± 0.455	

### Prognostic implications of SOX7, SOX9 and SOX10 expression in PCa

**Figure 4:**
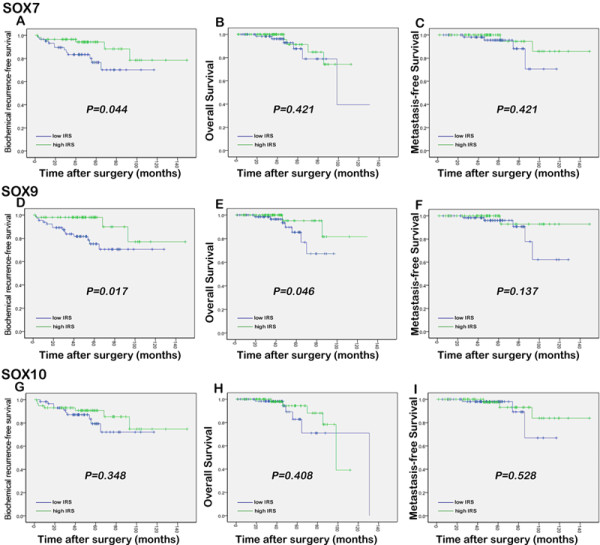
Kaplan-Meier survival curves of the biochemical recurrence-free survival, overall survival and metastasis-free survival for SOX7 (a, b and c, respectively), SOX9 (d, e and f, respectively) and SOX10 (g, h and i, respectively) expression in PCa.

The association of the expression levels of SOX7, SOX9 and SOX10 with the biochemical recurrence-free survival, overall survival and metastasis-free survival of PCa patients was analyzed using Kaplan-Meier method (Figure 4). The data indicated that there were no significant differences in overall and metastasis-free survival between high expression group and low expression group of SOX7 and SOX10 (all *p* > 0.05). Interestingly, the results by pairwise comparisons showed that there is a significant difference in the biochemical recurrence-free survival rates between patients with high SOX7 expression and low SOX7 expression (*p* = 0.044). In addition, the biochemical recurrence-free survival and overall survival rates of patients with high SOX9 expression were significantly lower than those with low SOX9 expression (*p* = 0.017 and 0.046), respectively. Furthermore, the multivariate analyses showed that the down-regulation of SOX7 (*P* = 0.010) and the up-regulation of SOX9 (*P* = 0.008) were independent predictors of shorter biochemical recurrence free-survival (Table 4).

**Table 4:** Prognostic value of SOX7 and SOX9 expression for the biochemical recurrence-free survival in multivariate analysis by Cox Regression

	**Hazard ratio (95% CI)**	***P* value**
SOX7 expression	0.81 (0.14-4.55)	0.010
SOX9 expression	0.20 (0.06-0.66)	0.008
Gleason Score	1.57 (0.71-3.45)	0.261
Pathological Stage	1.76 (0.54-5.72)	0.346
Serum PSA Levels	1.05 (0.98-1.12)	0.161

### Changes of SOX7, SOX9 and SOX10 expression in the progression of castration resistance in PCa

Nine xenograft tumor mouse models were established in this study and the xenograft tumor tissues were collected during androgen-dependent growth (AD), castration-induced regression nadir (ND), and castration-resistant regrowth (CR) stages (n = 3 for each stage). The tumor volumes were 3,620 ± 318 mm^3^, 697 ± 182 mm^3^, and 2,288 ± 301 mm^3^, and the median PSA values of the mice were 189.2 ng/mL, 4.3 ng/mL and 68.7 ng/mL for the AD, ND, and CR stages, respectively.

**Figure 5:**
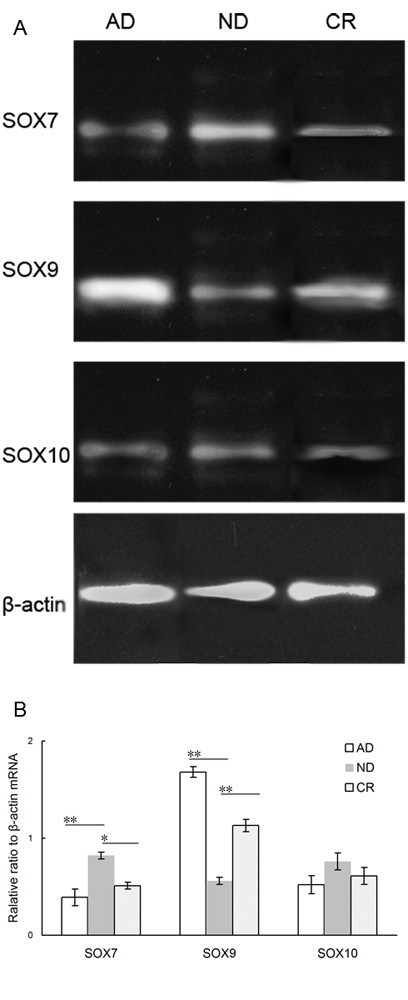
**Changes of SOX7, SOX9 and SOX10 mRNA expression levels in the progression of castration resistance in PCa.** (**a**) SOX7, SOX9 and SOX10 mRNA expression levels during androgen-dependent growth (AD), castration-induced regression nadir (ND), and castration-resistant regrowth (CR) stages detected by real-time quantitative RT-PCR. (**b**) The relative expression ratios of SOX7, SOX9 and SOX10 mRNA to β-actin mRNA during the AD, ND and CR stages, respectively. ‘*’ *P* < 0.05 compared with ND; ‘**’*P* < 0.01 compared with AD.

**Figure 6:**
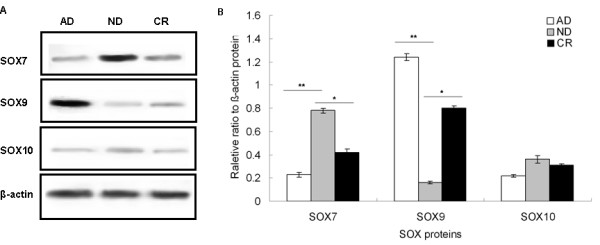
**Changes of SOX7, SOX9 and SOX10 protein expression levels in the progression of castration resistance in PCa.** (**a**) SOX7, SOX9 and SOX10 protein expression levels during androgen-dependent growth (AD), castration-induced regression nadir (ND), and castration-resistant regrowth (CR) stages detected by Western blot analysis. (**b**) The relative expression ratios of SOX7, SOX9 and SOX10 protein to β-actin protein during the AD, ND and CR stages, respectively. ‘*’ *P* < 0.05 compared with ND; ‘**’*P* < 0.05 compared with AD.

\Nextly, we evaluated the gene expression levels of SOX7, SOX9 and SOX10 at each stage using real-time quantitative RT-PCR. We found that the relative expression ratios of SOX7 mRNA to β-actin mRNA were 0.39, 0.82 and 0.51, the relative expression ratios of SOX9 mRNA to β-actin mRNA were 1.68, 0.56 and 1.13, and the relative expression ratios of SOX10 mRNA to β-actin mRNA were 0.52, 0.76 and 0.61 for the AD, ND, and CR stages, respectively (Figure 5). The changes of SOX7 and SOX9 expression in different stages had statistically significance, but that of SOX10 had not. These results indicated that SOX7 gene expression levels were significantly increased from the AD stage to the ND stage (AD vs. ND: *P* = 0.009), but decreased at the CR stage (*P* = 0.02). To be opposite, SOX9 gene expression levels were significantly decreased from the AD stage to the ND stage (AD vs. ND: *P* = 0.007), and also recovered at the CR stage (ND vs. CR: *P* = 0.01). Consistent with the trends at gene levels, the expression levels of SOX7 protein detected by Western blot analysis were significantly increased from the AD stage to the ND stage (AD vs. ND: *P* = 0.01, Figure 6), but decreased at the CR stage (*P* = 0.02, Figure 6). In contrast, the expression levels of SOX9 protein were significantly reduced from the AD stage to the ND stage (AD vs. ND: *P* = 0.006, Figure 6), and also recovered at the CR stage (ND vs. CR: *P* = 0.01, Figure 6).

## Discussion

PCa is the most frequently occurring cancer in men and advanced metastatic PCa is currently incurable. It is the great challenge of current basic and clinical research to identify novel molecular markers that could improve the tumor classification and prognostic stratification of PCa. The main findings of the present study are as following five points. Firstly, using gene microarray system, SOX7, SOX9 and SOX10 were identified as candidate genes which were differentially expressed in PCa compared with non-cancerous prostate tissues; Secondly, the de-regulation of SOX7, SOX9 and SOX10 genes and proteins in PCa tissues were further confirmed by real-time quantitative RT-PCR and immunohistochemistry analysis; Thirdly, the expression levels of SOX7, SOX9 and SOX10 in PCa tissues were related to the severity of the tumor malignancy; Fourthly, the down-regulation of SOX7 and the up-regulation of SOX9 were both independent predictors of shorter biochemical recurrence free-survival of patients with PCa; and finally, the down-regulation of SOX7 and the up-regulation of SOX9 at both gene and protein levels were observed during castration-resistant progression in xenograft tumor mouse models. These results suggest the SOX7, SOX9 and SOX10 play important roles in the pathogenesis and aggressiveness of PCa, and SOX7 and SOX9 especially be associated with the prognosis and be involved in the castration-resistant progression of PCa.

The main functions of the SOX gene family are as following [13-16]: Firstly, SOX genes regulate specification and differentiation of many cell types, such as neurogenesis, neural crest development, chondrogenesis, male sex gonad or respiratory epithelium development, melanocyte differentiation, and the differentiation of Paneth cells in the gut; Secondly, SOX genes within the same subgroup often share functional roles; Thirdly, SOX genes within the same subgroup can counteract the function of genes in another subgroup, and fourthly the same SOX gene can mediate different stages of development in one cell type and/or developmental processes in more than one cell type. Based on phylogenetic analysis of their HMG domains, SOX genes can be separated into subgroups A-J, A-H of which are represented in mouse and humans [17]. Because they are expressed in many tissues, it is not surprisingly that SOX genes are implicated in the etiology of many diseases and certain cancers. In the present study, we identified three SOX genes (SOX7, SOX9 and SOX10) as the genes of interest, because they were differentially expressed in PCa compared with adjacent benign prostate tissues using the gene microarray system (Table 2). The down-regulation of SOX7 and SOX10, and the up-regulation SOX9 genes and proteins in PCa tissues were further confirmed by the real time quantitative RT-PCR and immunohistochemistry analysis, when compared to the corresponding non-cancerous prostate tissues.

SOX7 gene belongs to SOX subgroup F, which also includes SOX17 and SOX18 [18]. It encodes a transcription factor that can both enhance and inhibit transcription. SOX7 is mainly implicated in parietal endoderm differentiation [19]. According to the previous studies, SOX7 mRNA is undetected in some human cancer cell lines including HeLa S3 (cervical cancer), K562 (chronic myelogenous leukemia), SW480 (colorectal cancer), etc., suggesting that SOX7 might be a tumor suppressor gene in these cancers [20]. In PCa, SOX7 mRNA and protein expressions were both shown to be down-regulated, which was consistent with the findings of this study and was found to be due to tumor-specific promoter hypermethylation present in PCa tissues and PCa cell lines/xenografts [10]. Interestingly, we further demonstrated that the expression level of SOX7 protein in PCa tissues with higher serum PSA level and metastasis were significantly lower than those with lower serum PSA level and without metastasis. SOX9, together with SOX8 and SOX10, belongs to SOX subgroup E [21,22]. It is a downstream effector of SRY, which in turn is dependent on the activity of androgens and the AR. There have been several studies on the involvement of SOX9 in PCa. In 2004, Drivdahl et al. [23] found that the elevated expression of SOX9 in PCa cell lines resulted in a decreased rate of cellular proliferation, cell cycle arrest in G0/G1, and increased sensitivity to apoptosis. In 2007 and 2008, Wang et al. [7,8] also demonstrated that SOX9 was expressed in PCa cells and was increased in relapsed hormonerefractory PCa. In 2010, Thomsen et al. [12] identified SOX9 as part of a developmental pathway that is reactivated in prostate neoplasia where it promotes tumor cell proliferation. All these previous studies were similar to the findings of our study. SOX10 has been implicated in the late stage of neural crest cell formation, maintenance of multipotency crest cells as stem cells and specification of derivative cell fates to Schwannian and melanocytic destinations [24-26]. Mutations in the SOX10 gene have been reported in a fraction of both primary and metastatic melanoma tumors [27]. The immunohistochemistry analysis has also been employed to map the expression of SOX10 in various human tissues and SOX10 has been suggested to be a specific and sensitive marker for melanocytic tumors [28]. However, the role of SOX10 in the development of PCa is not clear. From our investigation, SOX10 has been shown to be down-regulated and was associated with the high serum PSA levels and advanced pathological stage in PCa tissues.

An accurate prediction of the probability of disease recurrence is essential for proper therapy selection of PCa tissues. Thus, we investigated the prognostic significances of SOX7, SOX9 and SOX10 in the present study. Our results revealed that the expression of SOX7 and SOX9 in PCa correlated with biochemical recurrence-free survival. Patients with shorter follow-up time had tumors with a significantly lower expression of SOX7 and higher expression of SOX9, which indicated that tumors in patients with biochemical recurrence harbored SOX7 down-regulation and SOX9 up-regulation. In order to determine whether three SOX genes are involved in the progression to castration resistance PCa, we detected the changes of their expression in xenograft tumor mouse models. Our data showed the SOX7 down-regulation and the SOX9 up-regulation during the process of castration resistance, which suggested that the de-regulation of SOX7 and SOX9 may be one of the mechanisms responsible for the progression to castration resistance PCa. The exact roles of SOX7, SOX9 and SOX10 in the development of castration resistance are worth to be further investigation.

## Conclusions

In conclusion, our data offer the convince evidence that the de-regulation of SOX7, SOX9 and SOX10 may be associated with the aggressive progression of PCa. SOX7 and SOX9 may be potential markers for prognosis in PCa patients. Interestingly, the down-regulation of SOX7 and the up-regulation of SOX9 may be possible mechanisms for castration-resistant progression of PCa.

## Competing interests

The authors declare that they have no competing interests.

## Authors’ contributions

HH and WZ: participated in study design and coordination, analysis and interpretation of data, material support for obtained funding, and supervised study. GQ, QD and ZH: performed most of the experiments and statistical analysis and drafted the manuscript. SC: helped to translated and edit the paper. SW and CW: supported the microarray tissue slices and analysis the clinical related data. Other author: carried out the experiment and sample collection. All authors read and approved the final manuscript.

## Pre-publication history

The pre-publication history for this paper can be accessed here:

http://www.biomedcentral.com/1471-2407/12/248/prepub
